# A124 A SYSTEMATIC REVIEW OF THE EFFICACY OF ARTIFICIAL INTELLIGENCE IN IDENTIFYING BARRETT'S ESOPHAGUS NEOPLASIA

**DOI:** 10.1093/jcag/gwad061.124

**Published:** 2024-02-14

**Authors:** J Buttar, H Kim, M Byrne

**Affiliations:** The University of British Columbia Faculty of Medicine, Vancouver, BC, Canada; The University of British Columbia Faculty of Medicine, Vancouver, BC, Canada; The University of British Columbia Faculty of Medicine, Vancouver, BC, Canada

## Abstract

**Background:**

Barrett’s Esophagus (BE) is a known precursor for esophageal adenocarcinoma and requires frequent surveillance with esophagogastroduodenoscopy. Due to tumour heterogeneity and logistical demands on endoscopists, identification of BE dysplasia is difficult and random biopsies are riddled with sampling error. Artificial Intelligence (AI), in the form of computer-aided detection, has entered the endoscopic realm to improve BE dysplasia detection. This systematic review aims to evaluate its efficacy in BE screening.

**Aims:**

To survey the literature regarding the efficacy of machine learning tools in identifying BE dysplasia. The primary outcome was recognition of BE from a database of endoscopic images with histopathologic correlation utilizing a machine learning algorithm, with sensitivity and specificity reported.

**Methods:**

Using the PRISMA framework, MEDLINE, EMBASE and Compendex databases were searched from inception to Sept 1, 2023. Of 1915 articles identified, 35 were selected for full-text review. Two reviewers, JB and JK, independently completed the literature review and discrepancies were reviewed by the PI. After applying inclusion and exclusion criteria, 20 studies were included in the systematic review. The quality of studies was assessed using the Newcastle-Ottawa Scale.

Inclusion Criteria:

-Must include Barrett's esophagus and / or EAC (esophageal adenocarcinoma)

-Must include novel research (not a systematic review or MA)

-Requires use of endoscopic images

-English only, full text manuscripts

Exclusion Criteria:

-No prior surgery (esophagectomy)

-Does not include esophageal squamous cell carcinoma

**Results:**

Of the 20 articles selected, seven were published after 2021. All studies utilized a machine learning algorithm to aid in identification of BE dysplasia. Various imaging modalities were used, including white light imaging, narrow-band imaging, or volumetric laser endomicroscopy. A total of 3,886 patients were included with 5,605 images. Sensitivity ranged from 72 – 100%, specificity ranged from 64 – 94%. The overall quality of studies included was low.

**Conclusions:**

Surveillance of BE dysplasia remains a difficult and time-intensive task. Computer-aided detection of BE dysplasia demonstrates strong performance independent of the machine learning algorithm or imaging modality. Meta-analyses are required to illustrate heterogeneity and power to bolster these preliminary findings.

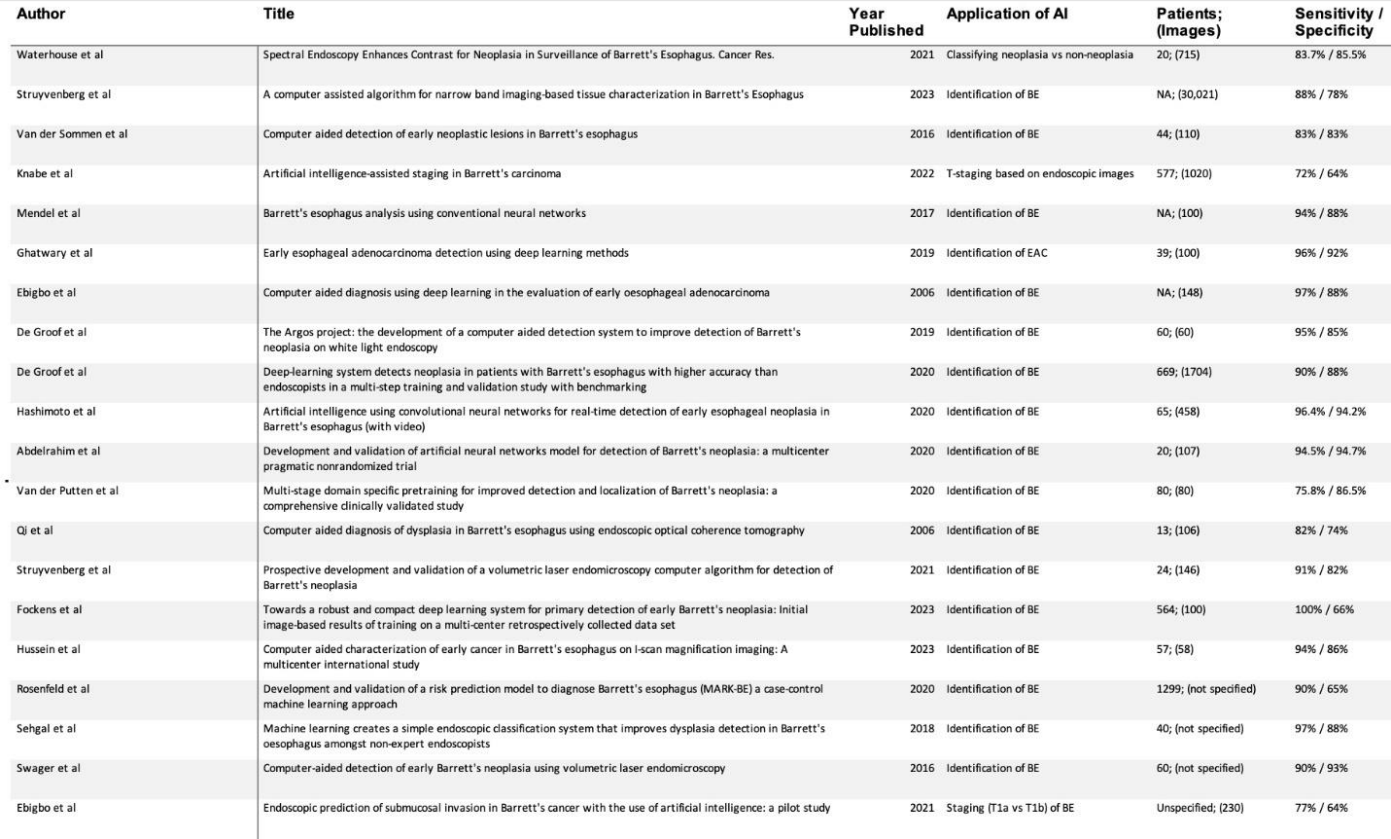

**Funding Agencies:**

None

